# Determinants of health seeking behavior of animal bite victims in rabies endemic South Bhutan: a community-based contact-tracing survey

**DOI:** 10.1186/s12889-019-6559-x

**Published:** 2019-02-27

**Authors:** Kinley Penjor, Tenzin Tenzin, Rinzin Kinga Jamtsho

**Affiliations:** 1 Dewathang Military Hospital, Department of Medical services, Ministry of Health, Thimphu, Bhutan; 2 Disease Prevention and Control Unit, National Centre for Animal Health, Department of Livestock, Thimphu, Bhutan; 3 Zoonosis Program, Department of Public Health, Ministry of Health, Thimphu, Bhutan

**Keywords:** Dog bite, Rabies, Post-exposure prophylaxis, Health-seeking behavior, Bhutan

## Abstract

**Background:**

Dog bites are the main source of rabies infection and death in humans, contributing up to 99% of all cases. We conducted a contact-tracing study to evaluate the health seeking and treatment compliance behaviors of people following potential exposure to rabies in rabies endemic south Bhutan.

**Methods:**

Using information from the rabies post exposure prophylaxis (PEP) register, animal-exposed victims who had visited five hospitals in south Bhutan between January and March 2017 were traced and further data were collected from them using a structured questionnaire. A snowballing technique was used to identify victims who did not seek PEP.The survey was conducted between April and June 2017. Logistic regression was performed to assess factors associated with PEP-seeking and compliance behavior by the victims.

**Results:**

Amongst 630 who reported to hospitals, 70% (444) of people could be traced and additional 8% (39) who did not seek PEP was identified through contact tracing. Therefore, a total of 483 people were interviewed. Seventy one percent (344/483) of exposure were due to animal bites of which 80% (365/455) were considered to be provoked incidents. Common reasons for not seeking health care included assumptions that risks of infection were minor if bitten by an owned or vaccinated dog. The victims who are male (OR: 0.36; 95% CI: 0.16–0.77) and educated (OR: 0.41; 95% CI: 0.17–0.96) were less likely to seek PEP, while those that experienced unprovoked bite (OR: 5.10; 95% CI: 1.20–21.77) were more likely to seek PEP in the hospitals. Overall, 82% of the victims sought PEP from the hospitals within 24 h after exposure. Eighty three percent completed the PEP course prescribed by the physician. The respondents living in urban areas (OR: 2.67; 95% CI: 1.34–5.30) were more likely to complete the prescribed PEP course than rural dwellers.

**Conclusions:**

There is high risk of rabies infection in southern Bhutan. It is critical to bridge knowledge gaps and dispel existing myths which will help to improve PEP seeking and compliance behavior of people exposed to rabies infection from animals. A risk-based advocacy program is necessary to prevent dog-mediated human rabies deaths.

**Electronic supplementary material:**

The online version of this article (10.1186/s12889-019-6559-x) contains supplementary material, which is available to authorized users.

## Background

Rabies remains an important zoonotic disease causing an estimated 59,000 human deaths globally, over 3.7 million disability-adjusted life years (DALYs) and 8.6 billion USD economic loss annually [1]. Dog bites are the main source of human deaths caused by rabies, contributing up to 99% of all rabies transmissions to humans [2]. Rabies disproportionately affects children below 15 years of age and economically disadvantaged people for whom accessibility to appropriate post-exposure prophylaxis (PEP) are limited [1, 3].

In Bhutan, rabies outbreaks were frequent and occurred throughout the country until 1992. The implementation of mass dog vaccination and dog population management program had drastically reduced the incidence of rabies in the interior parts of the country [4, 5]. Currently, rabies commonly occurs in the southern parts of the country that share border with India [6, 7]. However, sporadic outbreaks have occurred in some of the rabies free districts in Bhutan as a result of incursion of disease from the bordering areas, and risks establishing endemic transmission [8–10]. Between 2006 and 2016, 17 human deaths attributed to rabies, equating to a cumulative incidence of 0.23 per 10,000 population and over 7000 dog bites (1026 bites per 100,000 people annually) were reported in the country [4, 11]. The annual public health expenditure on human rabies PEP is approximately Nu. 9.3 million (USD 142,000) [12] and the cost is likely to increase with increasing dog bite incidence and higher level of awareness on rabies risk [4]. Rabies is a notifiable disease in Bhutan and all prevention and control activities are coordinated at the national level using One Health approach. The national rabies prevention and control plan 2017 and human rabies management guideline 2014 is being followed to guide rabies prevention and control activities in the country [13]. To further strategize the national efforts to achieve zero human deaths due to dog-mediated rabies before the global target of 2030 [14], Bhutan One Health Strategy Plan 2017–2021 has been developed and approved by the government to collaboratively implement rabies prevention and control in the country. Towards this end, one of the key tools to prevent human deaths from rabies is to improve accessibility and uptake of PEP following potential exposure, since most human deaths due to rabies occur from ignorance resulting in failure or delay in seeking PEP intervention from hospitals [3, 14].

This study evaluated the health seeking and compliance behaviors of people following potential exposure to rabies in south Bhutan using a contact-tracing questionnaire survey. The study findings are expected to inform strategies to improve PEP-uptake by ‘patients after exposure’ through targeted community-based education program and eventually achieve zero human deaths due to dog-mediated rabies in the country by 2023.

## Methods

### Study area

**Fig. 1:**
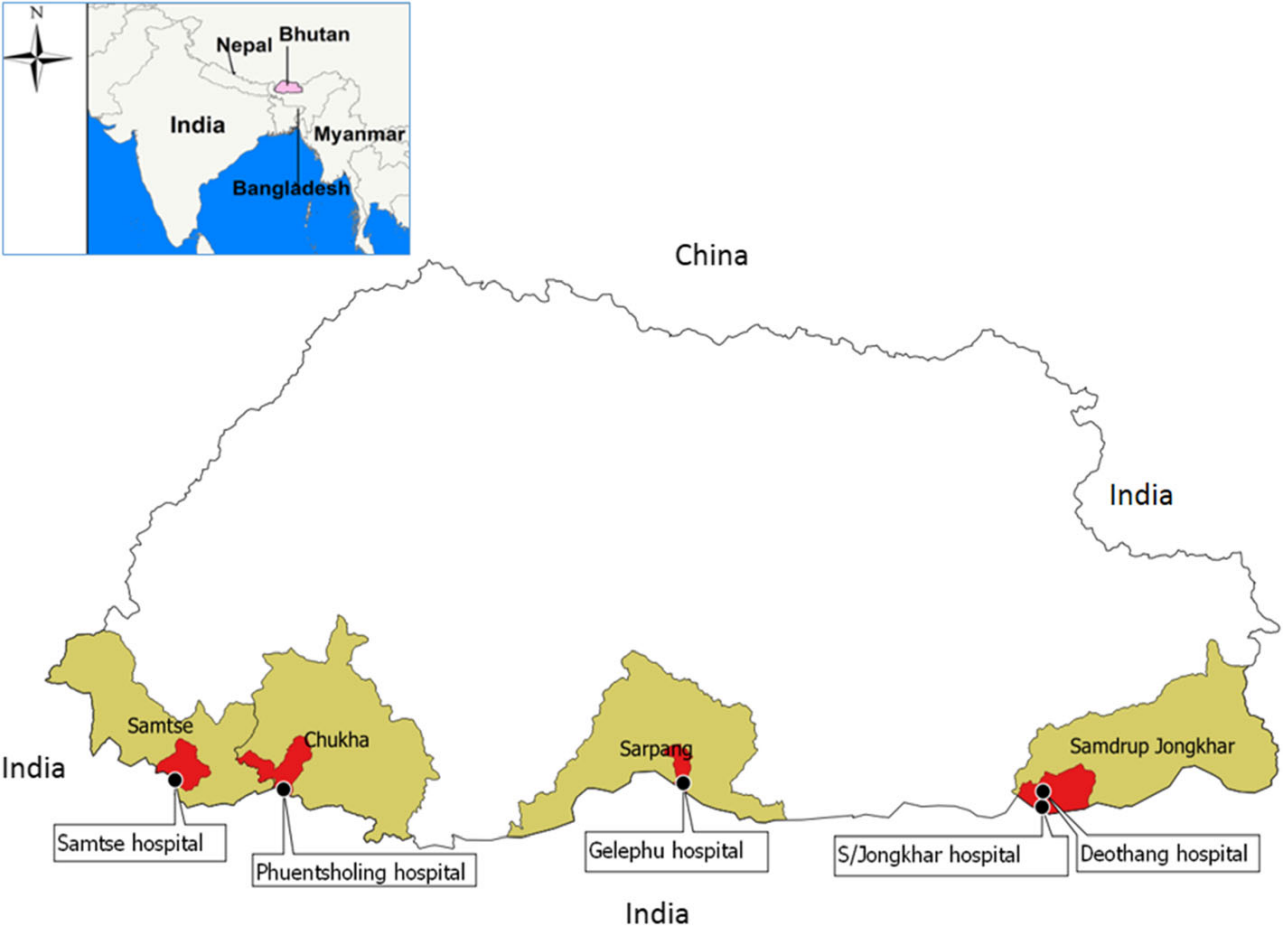
The selected study sites (hospital catchment area) in rabies endemic south Bhutan

The study was conducted in five catchment areas of government hospitals located in southern Bhutan where rabies is endemic - Phuentsholing hospital (Chukha district), Samtse hospital (Samtse district), Gelephu hospital (Sarpang district), Deothang hospital and Samdrup Jongkhar hospital (Samdrup Jongkhar district) (Fig. 1). These hospitals are the main healthcare facilities in their respective districts in southern parts of the country, where majority of the rabies outbreaks in dogs and the highest use of rabies vaccine in people occurs in the country [15–18].

These hospitals provides health care services including PEP to approximately 57,341 catchment population (Phuentsholing hospital: 27,658 people, Samtse hospital: 10,500, Gelephu hospital: 9858, Deothang hospital and Samdrup Jongkhar hospital: 9325). Therefore, only a very negligible number of rabies exposed people within these hospital catchment area might have seek PEP from village level Basic Health Unit (BHU) clinics when situation demand for their travel on business or due to other emergencies.

Bhutan is a Buddhist country located in South Asia and is administratively divided into 20 districts and 205 sub-districts. It has a population of about 0.73 million with an overall literacy rate of 71.4%. In Bhutan, all healthcare services including anti-rabies vaccine is provided free of costs to the population through a network of 30 hospitals and 210 BHUs. Typically, there is at least one hospital in each district and a BHU in each sub-district that provides healthcare services to the communities. All animal-bite victims that visit the hospitals/BHU to seek medical care are provided wound care and PEP for rabies if necessary after careful assessment by the clinical staff. An Updated Thai Red Cross Intradermal regimen (2–2–2-0-2) which requires four visits on day 0, 3, 7 and 28 post-exposure is being adopted in Bhutan. All types of vaccines including rabies vaccine are procured centrally by the health ministry and distributed to the hospitals and BHUs in the country. Thus, there are no parallel private healthcare facilities in Bhutan and private pharmacies are not allowed to sell rabies or any other vaccines [11, 15, 19–21].

### Data collection

A community-based questionnaire survey was conducted between April and June 2017. The following information was included in the questionnaire (see Additional file 1): socio-demographic characteristics of patients, awareness about rabies (including knowledge about susceptible animals, routes of transmission, signs of rabies in animal and rabies prevention and control measures), details on the nature of the exposure and PEP administration (vaccination status of biting dog/cats determined by review of vaccination card), additional people bitten by the same animal, dates of exposure and PEP administration, reasons for delay or not seeking PEP) and the costs incurred for visiting the hospitals for PEP treatment. If the circumstances of bite was associated with playing, feeding, touching its offspring, running in close proximity and handling injured animals, this was referred to as “provoked bite”. The questionnaire was piloted with 10 dog-bite patients and modified to improve clarity. PEP registers maintained in each hospital were used as the primary source of patient data. Using information from the PEP registers, animal-exposed victims that had visited hospitals between January and March 2017 were traced back and followed up in the community by telephone and personal visit for interview. Data about animal exposure and PEP details were retrieved from the PEP registers. The exclusion criteria used for data collection were: 1) any victims who could not be traced after 3 attempts to contact and interview; 2) any victims who declined consent to be interviewed, and 3) any victims who had died prior to follow-up. Interviewers were selected from the respective hospitals and trained on study protocol, questionnaires and data collection methods prior to administering the survey. The investigators supervised and coordinated the conduct of the field survey.

A snowballing technique was used to identify/trace people from the index patients for interview who had animal exposure but had not visited health centre for treatment. This is a non-probability sampling method in which dog-bite victims who had visited the hospitals for PEP recruited other bite victims who had not visited the hospitals within their community from among their acquaintances [22]. This was done to obtain comparative information related to socio-demographic factors that influenced the PEP-seeking behaviours of the individual.

For each patient contacted, the selected person was informed about the purpose of the study, that the participation was voluntary and data collected would be kept confidential. The interview was conducted with the victim himself/herself or with a supervising adult in the case of children less than 18 years of age, after obtaining written informed consent. The study was approved by Research Ethics Board of Health (REBH) vide approval letter No. REBH/Approval/2017/005.

### Data analysis

The data management and analysis was conducted using EpiInfo™ version 7.1.2.0 (Centers for Disease Control and Prevention (CDC), Atlanta, GA, USA) and Stata, version 14. Descriptive analysis was performed by calculating frequencies and percentages of variables of interest to investigate patterns of exposure. The factors associated with reporting to hospitals (PEP seeking) and completing the PEP regimen (PEP compliance) were assessed using logistic regression analysis. The risk factors investigated included: age group and gender, educational qualification, occupation, income level of the family, knowledge about rabies of the respondents, type of exposure (animal bite versus non-bite), ownership of the animal responsible for exposure (pet, stray and wild animal), vaccination status of biting animals (vaccinated vs non-vaccinated), location (rural vs urban), circumstances of exposure (provoked vs unprovoked), category of exposure (category I, II, III), rabies status of biting animal (normal, suspected, confirmed rabid) and distance in kilometers from the victims residence to the nearest hospital. Continuous (age of the respondents) and categorical variables (education, occupation, household income level, circumstances of animal exposures) were re-categorized for regression analysis. First a univariable logistic regression was conducted with “PEP sought vs PEP not sought” and “PEP completed vs PEP not completed” as an outcome with the above mentioned variables as predictors. Any variables with *p* < 0.25 were selected for the multivariable logistic regression model. The final models were built using forward stepwise elimination approach based on likelihood ratio tests and any variables with *p-*value of < 0.05 were considered significant and retained in the final model.

## Results

### Respondent socio-demographic characteristics

During January to March 2017, 630 people reported to five hospitals to seek rabies PEP (Gelephu: 219; Phuntsholing: 219; Deothang: 88; Samtse: 73; Samdrup Jongkhar: 31). Of 630 people, 444 (70%) could be traced back and interviewed. The remaining 186 people could not be traced/contacted despite repeated attempts (3 times through phone call) using the phone number recorded in the PEP treatment register maintained in the hospitals. The contact-tracing found additional 39 people that did not report to hospital to seek PEP following animal exposure. All 39 cases were either WHO category 2 (17) or 3 (21) except for one due to rat bite. Overall, 483 people were interviewed in this study, of which 55% were male and 45% were female. The median age of victims was 17 years (mean 23 years; range 1–83 years). All the recorded victims survived and no human rabies death was reported in Bhutan during the study period as well as during the whole year of 2017. Table 1 shows details of socio-demographic characteristics of the respondents.

**Table 1:** Socio-demographic characteristics of the participants (*n* = 483)

Variable	Number	Percent
Respondents from hospitals catchment area		
Gelephu	205	42
Phuntsholing	144	30
Samtse	68	14
Deothang	50	10
Samdrup Jongkhar	16	3
Gender		
Male	267	55
Age category (years)		
0-9 yrs	160	33
10-19 yrs	98	20
20–29 yrs	61	13
30-39 yrs	54	11
40-49 yrs	51	11
50 + yrs	55	11
Missing	4	1
Qualification of victims^a^		
No education	174	36.9
Education	309	64
Occupation of participants^b^		
Farmer	55	11
Student	189	39
Employed	108	22
Others	127	26
Missing	4	1
Type of settlements^c^		
Rural	123	25
Semi-urban	176	36
Urban	184	38
Household monthly income (USD)^d^		
< 154	248	51
154–308	158	33
309–462	43	9
463–615	16	3
> 615	9	2

^a^No education = respondents/victims who had not attained formal primary education level

Education = respondents/victims who had attained at least formal primary education level and above

^b^Employed = working in government or private sector; others = Housewives, pre-school children, religious persons

^c^Urban = those living within main district town; semi-urban = outskirts of main town; rural = villages

^d^1 USD = BTN 65 currency exchange rate at the time of conduct of this study

### Characteristics of animal exposure

Seventy one percent (344/483) of exposure resulted from animal bites of which 78% (270/344) were due to bites by dogs (175/344 by owned dogs and 95/344 by stray dogs) and 18% (61/344) by cats. The remaining 29% (139/483) of exposure was not bite-related, and was due to incidents such as scratches, handling rabid animal carcasses or coming into contact with secretions from rabid animals. The majority (76%, 365/483) of exposure was a provoked bite of WHO category 3 (67%, 325/483) type and more than 50% (237/458) of bites occurred on the lower limbs (Table 2).

**Table 2:** Characteristics of animal exposure

Variables	Number	Percent
Types of exposure		
Bites	344	71
Non-bites	36	7
Scratches	103	21
Ownership status of animal involved		
Pet dog	215	45
Pet cat	88	18
Stray dog	112	23
Stray cat	30	6
Livestock (handle/products)	24	5
Rat bite	10	2
Wild animal bite	4	1
Exposure circumstances		
Provoke	365	76
Un-provoke	90	19
Not applicable (others)^a^	28	6
WHO exposure category		
Category 1	22	5
Category 2	136	28
Category 3	325	67
Bite site (*n* = 458)		
Head	20	4
Upper limbs	184	40
Trunks	17	4
Lower limbs	237	52
Vaccination status of dog (*n* = 445)		
Vaccinated	145	33
Unvaccinated	163	37
Unknown	137	31

^a^Dairy product consumption and handling of rabid animal/carcasses

### Knowledge and awareness on rabies

**Fig. 2:**
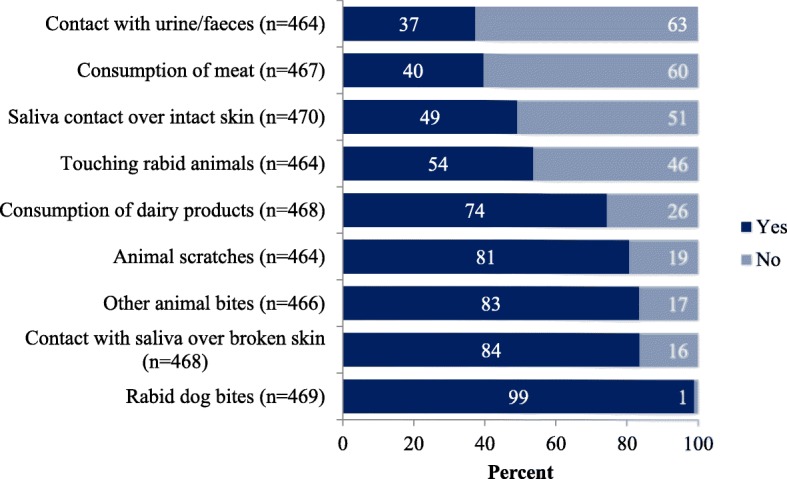
Knowledge of survey respondent on routes of rabies virus transmission

The majority of the respondents (98%, 471/483) had heard of rabies from various sources including health workers (55%), friends and relatives (62%), media (41%), school (32%), livestock officials (10%) and internet (14%). Ninety nine percent (*n* = 469) of the respondents knew that rabies is transmitted from dog bites, 91% (*n* = 450) knew signs of rabies in animals, 93% (*n* = 461) knew rabies is a fatal disease and 92% (*n* = 450) were able to state some preventive measures. However, 49% of the respondents believed that contact with secretions from a rabid animal over intact skin and touching of the animal (54%) will transmit rabies (Fig. 2). In terms of first aid after animal exposure, 51% (241/470) of the respondents mentioned that they washed bite wound with soap and water while 24% (116/471) had not done anything to the exposed site.

### Determinants of health seeking and compliance behavior

**Fig. 3:**
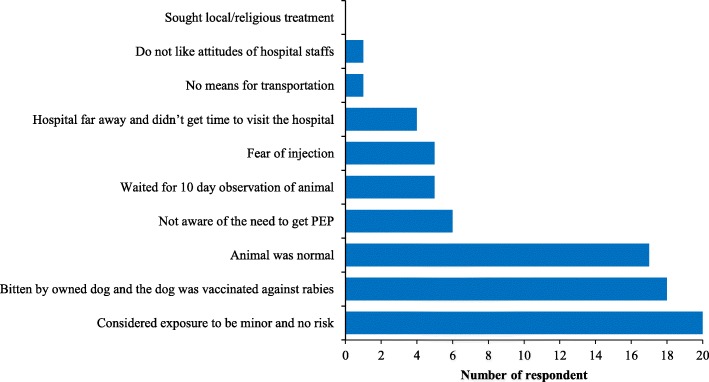
Reasons for not seeking PEP following animal exposures (*n* = 39)

The contact-tracing survey found that 8% (39/483) of the victims did not report to hospitals following animal exposure. The most common reason for not seeking PEP treatment were assumption by the victim that risk was minor due to bite by owned and vaccinated dog and the biting animal was healthy and normal (Fig. 3). Univariable logistic regression analysis indicated seven factors associated with PEP seeking behavior (Table 3).

**Table 3:** Univariate analyses of factors associated with likelihood of animal exposed victims seeking rabies PEP

Variable	Unadjusted OR	95%CI	*P*-Value
Age category (years)			
< 15	1.00	–	–
> 15	1.50	0.77–2.89	0.23
Gender			
Female	1.00	–	–
Male	0.34	0.16–0.74	0.006
Place types			
Rural	1.00	–	–
Semi-urban	0.59	0.22–1.59	0.29
Urban	0.45	0.17–1.15	0.09
HH income level^a^			
High	1.00	–	–
Medium	0.95	0.38–2.41	0.92
Low	2.07	0.70–5.42	0.14
Educational qualification^b^			
No education	1.00	–	–
Education	0.37	0.16–0.86	0.02
Exposure types			
Bites	1.00	–	–
Non-bites	0.49	0.25–0.95	0.04
Exposure circumstances			
Provoke	1.00	–	–
Un-provoke	4.96	1.17–21.0	0.03

^a^High income = above 450 USD; medium income = 150 to 450 USD; low income = below 150 USD

^b^No education = respondents/victims who had not attained formal primary education level

Education = respondents/victims who had attained at least formal primary education level and above

Multivariable logistic regression analysis demonstrated that male (OR: 0.36; 95% CI: 0.16–0.77) and educated victims (OR: 0.41; 95% CI: 0.17–0.96) were less likely to seek PEP while the victims with unprovoked exposure incidents are more likely to seek PEP in the hospitals (OR: 5.10; 95% CI: 1.20–21.77) (Table 4).

**Table 4:** Final multivariate logistic regression model of factors associated with likelihood of animal exposed victims seeking PEP

Variable	Adjusted OR	95% CI	*P*-value
Sex			
Female	1.00	–	–
Male	0.36	0.16–0.77	0.009
Educational qualification			
No education	1.00	–	–
Education	0.41	0.17–0.96	0.04
Exposure circumstances			
Provoke	1.00	–	–
Un-provoke	5.10	1.20–21.77	0.028

**Fig. 4:**
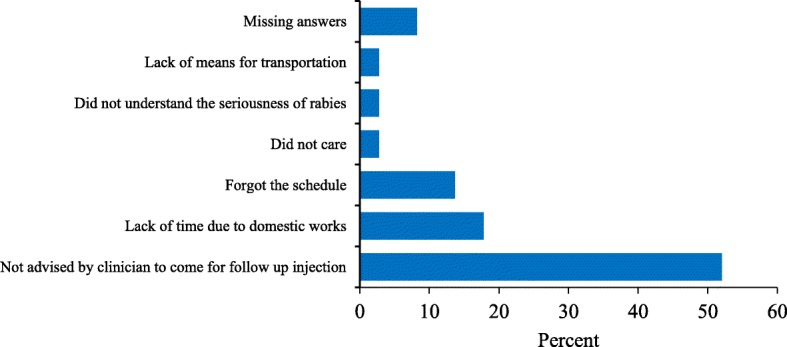
Reasons for not completing the PEP course in rabies endemic areas of Bhutan (*n* = 73)

**Fig. 5:**
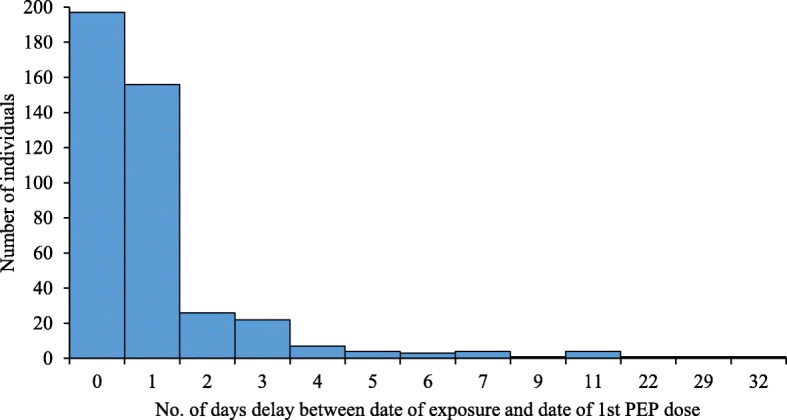
Time delay between date of animal exposure and date of first post exposure vaccination amongst animal exposed victims (*n* = 427)

Based on the contact-tracing interview, 83% (359/432) of the animal-exposed victims who were administered rabies PEP completed the regimen prescribed by the physician while 17% (73/432) did not complete the recommended course for various reasons. The majority of patients failing to complete the treatment (52%) stated that they were not informed by the clinicians about follow up injection (Fig. 4). Univariate logistic regression analysis indicated ten factors associated with patient’s compliance with the prescribed PEP course (Table 5). The respondents living in the urban area were more likely to complete the prescribed PEP course than rural dwellers (OR: 2.67; 95% CI: 1.34–5.30). Overall 82% (352/427) of the animal exposed victims sought PEP from the hospitals within 24 h; 46% (197/427) had received PEP on the same day and 36% (156/427) on the following day (Fig. 5).

**Table 5:** Univariate analyses of factors associated with likelihood of completing PEP course

Variable	Unadjusted OR	95% CI	*P*-Value
Age (years)			
< 15	1.00	–	–
> 15	0.68	0.40–1.14	0.149
Sex			
Female	1.00	–	–
Male	1.44	0.87–2.39	0.155
Respondents living area (place types)			
Rural	1.00	–	–
Semi-urban	1.12	0.62–2.02	0.71
Urban	2.57	1.30–5.09	0.007
Educational qualification			
Education	1.00	–	–
No education	0.46	0.28–0.77	0.003
Occupation			
Employed	1.00	–	–
Farmer	0.45	0.20–0.98	0.044
Student	1.16	0.59–2.29	0.671
Others	1.09	0.52–2.27	0.816
Heard Rabies?			
No	1.00	–	–
Yes	2.92	0.83–10.23	0.095
HH income level^a^			
High	1.00	–	–
Medium	1.27	0.60–2.70	0.53
Low	1.56	0.76–3.17	0.22
Exposure species			
Others	1.00	–	–
Pet animal	0.37	0.01–1.24	0.109
Stray animal	0.58	0.16–2.09	0.406
WHO exposure category			
Cat I	1.00	–	–
Cat II	0.93	0.31–2.75	0.89
Cat III	1.84	0.65–5.27	0.25
Distance to PEP centers			
< 5kms	1.00	–	–
> 5kms	1.63	0.96–2.76	0.068

Note: ^a^High income = above 450 USD; medium income = 150 to 450 USD; low income = below 150 USD

**Fig. 6:**
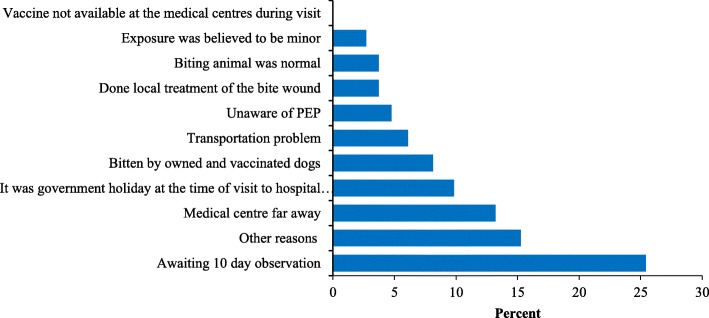
Reasons for delay in seeking PEP amongst survey respondents beyond 24 h of exposure (*n* = 75)

The main reasons for delay (beyond 24 h) in PEP seeking included individual decision to observe animal behavior before visiting health centers for PEP and long distances or transportation problem (Fig. 6). Majority of respondents (80%, 386/438) lived within 1 to 10 km of hospitals/BHU and more than 70% (307/439) used either private car or taxis. The average time spent by a victim for to and fro journey to hospitals/BHU and waiting time for consultation in hospital is 3.55 h and 2.55 h for first and subsequent follow up visits respectively. The abstract of this study.

## Discussion

We conducted a contact-tracing survey to understand the rabies PEP seeking behavior and compliance of people in terms of probability of returning for subsequent PEP injection after potential exposure to rabies. The results indicate that animal exposure with potential rabies risk remains an important public health problem in southern districts in Bhutan. For instance, more than 600 people have visited five hospitals in the south rabies endemic areas of Bhutan for PEP during the study period (January–March 2017), highlighting health risk as well as economic implications of the problem. Previous studies have also demonstrated high patient throughput for rabies PEP in these study areas due to frequent cross-border outbreak of rabies in animals [16–18].

Although majority of (71%) of the exposure were associated with animal bites, the respondents were not able to differentiate whether they were bitten by a rabid animals or healthy animals during interview nor is such data maintained in the hospital PEP register. As human rabies exposures depend on the incidence of rabid dogs and the rate at which rabid dogs bite people, it is important to maintain good record of these information in the hospitals to clearly delineate between exposures due to confirmed/suspected rabid animals and healthy animals. Such information would help to understand the proportion exposed to rabid animal and aid in PEP decision making. Thus, it is important to strengthen the surveillance system and implement integrated bite case-management, particularly in rabies high risks districts in Bhutan in line with the latest WHO recommendations [14].

Our study demonstrated that the majority of the respondents had heard about rabies and its prevention which is in concordance with reports of previous studies in Bhutan [10, 16, 23] and other studies in south Asian and African countries [24–26]. Higher level of community awareness on rabies could be attributed to endemic transmission and frequent reports of outbreaks in the study areas combined with regular advocacy on rabies conducted by the government [27]. However, there exist some knowledge gaps amongst the respondents. For instance, many of the respondents believed that rabies can be transmitted via contact with urine and feces of animals suspected of having rabies (37%), contact with saliva over intact skin (49%) or by just touching of rabid animals (54%). Such misconceptions about rabies give rise to undue fear and anxiety in people and often result in unnecessary PEP administration by health workers, despite the risk being low or negligible [4, 10, 17, 18, 23]. In this study, nine rat bites and four exposure incident involving consumption of dairy products or contacts over intact skin were administered PEP. On the other hand, many respondents also believed that scratches caused by animals and contact with rabid dog saliva over broken skin do not constitute risk of rabies transmission (Fig. 2). Therefore, it is important to prioritize awareness campaigns and educate both the public and health workers with correct information on rabies and its transmission to improve PEP-seeking behavior by the people, and rabies risk assessment by the health workers. Such interventions would reduce misconceptions about rabies transmission, thereby reducing unnecessary public concern and PEP administration, as well as prevent deaths due to rabies [23].

Rabies is a fatal disease and timely wound washing and PEP following exposure is vital for rabies prevention. In Bhutan, PEP is provided free of cost to animal-bite victims by the government through a network of 240 health centers. However, our study found that some people (8%) did not visit hospital for medical advice following animal bites. Several reasons were mentioned by the victims for not seeking PEP, including beliefs that risk of rabies infection was very low if the animal-bite wound was minor and they were bitten by owned and vaccinated dogs, and the biting animal was normal (Fig. 3). However those assumptions were incorrect as all the exposure occurred in rabies high risks areas and were found to be either WHO category 3 or 2 except for nine rat bite cases for which no PEP is recommended as per WHO or national guidelines. Studies in other rabies endemic countries have found similar or much higher proportion of rabies exposed victims (Sri Lanka: 7.3%, Tanzania: 20%, Guangdong province in China: 67% and Philippines: 92%) who did not seek PEP following potential rabies exposure from animal-bite [28–32]. Awareness level about rabies, socio-economic status, accessibility and availability of PEP are some of the major factors associated with PEP seeking behavior by the victims following potential rabies exposures [1, 3, 33, 34].

This study showed that the majority (82%) of the victims had visited the hospitals to receive PEP within 24 h of exposure. As discussed above, this could be due to a high level of awareness about rabies amongst people in Bhutan [10, 16, 23]. Administration of PEP immediately after exposure is critical to saving humans from developing rabies. However, some of the victims (17%) had not completed the prescribed PEP course. Lack of advice by medical staff to come for follow up injections, time constraints due to domestic work, and forgetting the schedule were some of the reasons mentioned by victims for not completing the PEP course (Fig. 4). Moreover, since pet dogs and cats were involved in the majority of bite incidents, the victims could have discontinued the course when the biting animal remained healthy after 10 days of observation. The PEP compliance rate is very high in Bhutan when compared to those reported from countries like Sri Lanka (10%) and Ethiopia (57%) [28, 30, 35]. The high compliance rate in Bhutan could be due to high level of awareness on rabies and also due to easy accessibility and availability of free rabies vaccine in the hospitals.

Logistic regression analysis demonstrated that the victims residing in urban and semi-urban areas are more likely to complete the prescribed PEP course than rural residents. Good compliance amongst urban residents compared to victims from the rural areas could be due to better understanding of rabies risk because of frequent outbreak of rabies in bordering towns and also high level of awareness on rabies amongst urban people. In addition, the close proximity of the hospitals in the urban areas could have improved the PEP adherence as observed in other studies elsewhere [30, 35]. In our study area, all hospitals were located in the center of the town providing easy access for residents living in town, while the people from rural areas within these hospital catchment had to travel repeatedly (four visits to receive the full four-injection course) to the hospital. The average time duration spent for availing PEP by the victims was 3.55 h and 2.55 h for first and subsequent visits, respectively. Thus, some of the victims from the rural areas could have failed to complete the course due to loss of time for work and limited access to transportation [17]. Non-compliance to prescribed PEP (incomplete PEP course) up to 50% have been identified in previous studies but no human rabies mortality due to incomplete PEP course was observed in Bhutan [17, 18]. This could be due to bite by healthy dogs for which also a PEP is given as per the national guidelines for management of rabies owing to prevalence of rabies in southern parts of the country [36].

There are few limitations in this study. Our study estimated the number of exposed victims seeking rabies PEP based on the number of cases who were registered in the health center, while the number of people who did not seek PEP was obtained through interview of index case. The study design could potentially bias the comparison of two groups of victims. It is likely that we underestimated the proportion of exposed cases as well as those not-seeking PEP, and overestimated the proportion of cases who visited health center because the number of bites cases in the denominator may be incomplete. This could be improved by conducting house-house data collection and in-depth case investigation. However, in our study settings where disease awareness is found to be fairly high coupled with easy and free access to vaccine, we expect the underreporting to be very minimal.

## Conclusions

Our study findings provide valuable insights on knowledge and perceptions that influence health seeking and PEP compliance behavior of animal exposed victims in rabies high risk region of Bhutan. The findings can inform in formulation of targeted educational and awareness program on rabies, particularly addressing on the incorrect assumptions (owned and vaccinated dog will not transmit rabies) and source and route of rabies transmission. A well planned, focused and evidence based strategies should be implemented in the communities to reduce undue fear of rabies and to optimize uptake and use of PEP by the animal exposed victims. It is critical that at-risk groups (males and rural section of population) be prioritized for awareness education program to improve their treatment seeking and compliance behavior to prevent human deaths from rabies. Such targeted interventions will facilitate country to achieve zero human death from dog- mediated rabies before the global target of 2030.

## Additional file

Additional file 1: Study Questionnaire. (DOCX 7227 kb)
