# Supplementary material for: Determinants of health seeking behavior of animal bite victims in rabies endemic South Bhutan: a community-based contact-tracing survey
Source: BMC Public Health. 2019 Feb 27;19:237. doi: 10.1186/s12889-019-6559-x (PMC6391763; doi:10.1186/s12889-019-6559-x)
Supplement: Supplementary file 1 — Study Questionnaire. (DOCX 7227 kb) [file 12889_2019_6559_MOESM1_ESM.docx]

**QUESTIONNAIRE**

**Determinants of health seeking behavior of animal bite victims in rabies endemic south Bhutan: A community-based contact-tracing survey**

**General Instruction:**

- This questionnaire consists of 5 parts- I, II, III, IV and V
- All questionnaire parts will need to be administered for all “PEP case” who presented to Health centres and initiated rabies PEP
- This same questionnaire (except some question in part III and all in Part IV) will also be administered for any exposed contacts identified in the community who did not seek PEP in the Hospitals through contact tracing
- The questionnaire shall be completed using data from ARV records maintained in the Hospitals and through interview of animal exposed case in the community, traced using information from ARV register
- Interviewer will select one or more response, whenever relevant
- This questionnaire shall be completed by the Investigators or the designated health or veterinary staff who will be trained by the Investigators
- The quality of information recorded in terms of completeness, correctness and timeliness will be assured by the investigators and designated health staff
- The investigators shall be responsible for privacy and safe custody of the respondents’ information and the completed questionnaires

**Operational Definition:**

- **Post Exposure Prophylaxis (PEP) Case** is defined as animal exposed victims who reported to the selected hospitals for rabies risk assessment and PEP treatment
- **Animal Exposure**- is defined as bites, scratches, nibbled or licks by any type of animals including wild animals except by reptiles or birds, or a person handling carcass of a suspected or confirmed rabid animal, or those who consumed animal products like meat, milk, dairy products from a suspected or confirmed rabid animal
- **Post exposure prophylaxis (PEP)**- is a preventive treatment against rabies consisting of local treatment of the wound, anti-rabies vaccine (ARV) administration with or without rabies immunoglobulins (RIGs)

**Questionnaire**

| Survey No.  (3-digit letter of hospital followed by 3-digit number for a patient) *PNG for Phuentsholing; GEL for Gelephu; SAM for Samtse, DEO for Deothang)* | \|  \|  \|  \|  \|  \|  \| \| --- \| --- \| --- \| --- \| --- \| --- \| |
| --- | --- | --- | --- | --- | --- | --- | --- |
| Date of survey  DD/MM/YY | \|  \|  \|  \|  \|  \|  \| \| --- \| --- \| --- \| --- \| --- \| --- \| |
| Recorded by: |  |

**Part I: Respondents’ information**

| 1.1 Name of the victim: |  |
| --- | --- |
| 1.2.Relationship of the respondent with the victim: *(if the victim is a minor and if you are interviewing parents/guardian of a minor)*  ***Minor:*** *those victims who are under 12 years of age will be considered as Minor in this survey.* | 1. Father 2. Mother 3. Brother 4. Sister 5. Guardian |
| 1.3 Age of the victim (rounded to years): |  |
| 1.4 Age of the respondent *(age of the Parents/guardian will be noted if the victim is a minor):* |  |
| 1.5 Gender of victim: | 1. Female 2. Male |
| 1.6 Gender of respondent *(gender of the Parents/guardian will be noted if the victim is a minor):* | 1. Female 2. Male |
| 1.7 Contact No: |  |
| 1.8 Name of Resident village/town *(mention the location & and also nearest identifiable location):* |  |
| 1.9 Type of settlement: | 1. Rural 2. Semi-urban 3. Urban |
| 1.10 Location of household *(if the lat long data collection is not possible, the nearest identifiable place will be used to locate the areas using Google Earth software)* | Latitude:  Longitude: |
| 1.11 Gewog/sub-district: |  |
| 1.12 District: |  |
| 1.13 Educational qualification of victim: | 1. No education 2. Non-Formal Education 3. Primary level 4. Lower Secondary 5. Higher Secondary 6. University graduate 7. Post-graduate 8. Buddhist studies 9. Others |
| 1.14 Educational qualification of respondent *(qualification of the Parents/guardian will be noted if the victim is a minor):* | 1. No education 2. Non-Formal Education 3. Primary level 4. Lower Secondary 5. Higher Secondary 6. University graduate 7. Post-graduate 8. Buddhist studies 9. Others |
| 1.15 Occupation of victim: | 1. Farmer 2. Student 3. House wife 4. Government employee 5. Businessman 6. Private/corporate sectors employee 7. Pre-school 8. Military 9. Monks/Nuns 10. Others |
| 1.16 Occupation of respondent *(occupation of the Parents/guardian will be noted if the victim is a minor):* | 1. Farmer 2. Student 3. House wife 4. Government employee 5. Businessman 6. Private/corporate sectors employee 7. Pre-school 8. Military 9. Monks/Nuns 10. Others |
| 1.17 Monthly Household income: | 1. less than Nu.5000 2. 5000-10,000 3. 10,000- 15,000 4. 15,000- 20,000 5. 20,000- 30,000 6. 30,000- 40,000 7. above 40,000 |

**Part II: Knowledge & Awareness on Rabies**

| **Question** | **Response** | | | |
| --- | --- | --- | --- | --- |
| 2.1 Have you heard of Rabies? | 1. Yes 2. No   *If the answer is* ***No, go to part III*** | | | |
| 2.2 If Yes, what is the source of rabies information? | 1. Health workers 2. Livestock officials 3. Friends/Relatives 4. Media (Kuensel/ BBS /Radio etc.) 5. Schools 6. Internet 7. Others | | | |
| 2.3. Do you know that following animal will get rabies? *(Tick the appropriate box)* | **Animal species** | Yes | No | Not Sure |
|  | 2.3.1. Dog |  |  |  |
|  | 2.3.2. Cat |  |  |  |
|  | 2.3.3. Domestic livestock |  |  |  |
|  | 2.3.4. Wild animals |  |  |  |
|  | 2.3.5. Rats/Rodents |  |  |  |
| 2.4. Do you know that following routes will transmit rabies virus (from rabid animals)? *Evaluate the respondents by asking whether human will get rabies through the following routes (Tick the appropriate box)* | **Routes** | Yes | No | Not sure |
|  | 2.4.1.Dog bites |  |  |  |
|  | 2.4.2.Other animal bites |  |  |  |
|  | 2.4.3. Animal scratches |  |  |  |
|  | 2.4.4. Contact with saliva over an intact skin |  |  |  |
|  | 2.4.5. Contact with saliva over broken skin |  |  |  |
|  | 2.4.6. Contact with urine/faeces |  |  |  |
|  | 2.4.7. Consumption of dairy products |  |  |  |
|  | 2.4.8. Consumption of cooked meat |  |  |  |
|  | 2.4.9. Touching rabid animal |  |  |  |
| 2.5 Evaluate the respondents by asking whether the respondents have knowledge on clinical signs and symptoms of rabies in animals (Dogs)  *(If the respondent answers aggressive behaviour, excessive salivation, barking, biting, then record as “Yes”)* | 1. Yes 2. No | | | |
| 2.6 How serious is rabies? *Ask whether the respondent have knowledge about the serious nature of rabies-*  *Ask the participant if rabies can kill people/animals - If the respondent answers 100% fatal, then record as “Yes”).* | 1. Yes 2. No | | | |
| 2.7 Whether the respondents have knowledge on the prevention & control of rabies  *(If the respondent answers wound washing, visit to hospital for PEP and vaccination and population control of dogs, then record as “Yes”)* | 1. Yes 2. No | | | |

**Part III: Animal bite details**

| **Question** | **Response** |
| --- | --- |
| 3.1 Date of animal exposure *(To be extracted from ARV register or else from respondent during interview)* | Date in DD/MM/YY   \|  \|  \|  \|  \|  \|  \| \| --- \| --- \| --- \| --- \| --- \| --- \| |
| 3.2 Date of ARV injection  *(To be extracted from ARV register)*  **NA for exposed case who did not seek PEP* | Date in DD/MM/YY   \|  \|  \|  \|  \|  \|  \| \| --- \| --- \| --- \| --- \| --- \| --- \|   **If the ARV was given on the same day of exposure, go to 3.4** |
| 3.3 If ARV was not initiated on the same day of exposure, list the reason/s?  **NA for exposed case who did not seek PEP* | 1. Not aware of the need to get PEP 2. ARV was not available at the hospital during the   visit   1. Bitten by owned dog and the dog was vaccinated   against rabies   1. Hospital far away and didn’t get time to visit the   hospital   1. Sought local/religious treatment 2. Animal was normal 3. Waited for 10 days observation 4. Considered the exposure to be minor and no risk 5. No money for transportation for immediate visit 6. The exposure day was government holiday 7. The clinician in the hospital ask me to come on the other day |
| 3.4 Type of exposure | 1. Bite wound with bleeding 2. Bite without bleeding 3. Scratches 4. Licks on the mucus membrane   or broken skin  contacts on the intact skin   1. Nibbling on skin 2. Consumption of raw milk/meat 3. Consumption of boiled milk/cooked meat 4. Handling of rabies suspected animal 5. Contacts with rabid humans 6. Family members of rabies case in animals |
| 3.5 Animal species responsible for the exposure? | 1. Pet dog 2. Pet cat 3. Stray dog 4. Stray cat 5. Rat/rodent 6. Livestock (cattle/goat/sheep/pig/horse) 7. Wild animals |
| 3.6 If the bite/scratch is by pet dog/cat, is it your own or neighbours? | 1. Own 2. Neighbour |
| 3.7 Where did the bite incident took place? | 1. At my house 2. At neighbours’ house 3. In the street 4. Institute/ campus |
| 3.8 What is the vaccination status of biting dog/cat? | 1. Vaccinated 2. Not vaccinated 3. Don’t know |
| 3.9 Was the dog responsible for bite ear notched?  *(Permanent identification mark applied to dogs vaccinated and sterilised in Bhutan)* | 1. Yes 2. No 3. Don’t know |
| 3.10 What were the external circumstances leading to bite? | 1. Playing with dog 2. Disturbance of dog while eating/nursing puppies 3. Surprising the dog 4. At the time of Vaccination/Medication (handling) 5. Teasing the dog 6. Interfering the dog while fighting 7. Kicking the dog 8. Disciplining the dog 9. Stepping on dog while walking 10. Cuddling the dog 11. Feeding the dog 12. Playing near the dog 13. Passing the dog while walking 14. Looking at the dog (eye to eye contact) 15. Cuddling the puppies 16. Screaming while approaching the dog 17. While running away from dog 18. Others |
| 3.11 Anatomical site of bite on the body?  *(Skip in non-bites exposures)* | 1. Head/neck/face 2. Upper limbs 3. Trunks/Genitalia 4. Lower Limbs (Thigh, leg and foot |
| 3.12 How many bite wounds have been caused *(Skip in non-bites exposures)* | 1. ☐ Single bite wound  2. ☐ Multiple bite wound  3. ☐ Scratches only without bleeding |
| 3.13 What was the category of exposure risk as assigned by Clinician?  *(Extract from ARV Register)*  **NA for exposed case who did not seek PEP* | 1. Category I 2. Category II 3. Category III |
| 3.14 What was the correct category of exposure risk?  *(To be determined from the interview by investigators)* | 1. Category I 2. Category II 3. Category III |
| 3.15 What were the clinical signs of biting animal? | 1. Apparently normal 2. Unprovoked aggression (Attempting to bite and grip people, animals, or objects) 3. Excessive salivation 4. Abnormal vocalization 5. Aimless movement/Running/wandering 6. Paralysis 7. Biting many other people/animals 8. Restlessness 9. known to have been bitten by rabid animal 10. Unexplained dullness/lethargy |
| 3.16 What happened to the biting animal? | 1. Dog still alive 2. Killed 3. Died 4. Disappeared 5. Dog was found on the same area of bite 6. Don’t know |
| 3.17 Was the biting animal confirmed for rabies? | 1. Yes 2. No 3. Don’t know |
| 3.18 If yes, how was it confirmed? | 1. Clinical signs of rabies 2. Laboratory test 3. Don’t know |
| 3.19 Has this biting animal bitten other people in the area | 1. Yes 2. No 3. Don’t know |
| 3.20 If the dog has bitten other people in the area, do you know whether they have visited hospital/BHU? | 1. Yes 2. No 3. Don’t know |
| 3.21 What have you done immediately to the exposed site before you visited the hospital/BHU? | 1. Nothing 2. Wound washing with water only 3. Wound washing with soap and water 4. Applied local herbs/medicine 5. Applied antiseptics after wound washing 6. Applied antiseptics without wound washing |
| 3.22 Did the hospital staff wash your wound with soap and water/ anti-septic solutions?  **NA for exposed case who did not seek PEP* | 1. Yes 2. No 3. Don’t remember |
| 3.23 Did you receive anti-rabies vaccine at hospital?  *(Extract from ARV Register)*  **NA for exposed case who did not seek PEP* | 1. Yes 2. No |
| 3.24 If Yes, did you complete the full PEP course  **(0,3,7 & 28 day injection)**  **NA for exposed case who did not seek PEP* | 1. Yes 2. No |
| 3.25 If yes, did you receive full PEP course or not?  **NA for exposed case who did not seek PEP* | 1. Yes 2. No |
| 3.26 If no, which schedule did you miss?  *(Extract from ARV Register; day 14 if IM was given)*  **NA for exposed case who did not seek PEP* | 1. Day 0 2. Day 3 3. Day 7 4. Day 14 5. Day 28 |
| 3.27 If you have not completed the PEP course, what were the reasons?  **NA for exposed case who did not seek PEP* | 1. Lack of time from domestic works 2. Not advised by clinician to come for follow up injection 3. Forgot the schedule 4. Did not care 5. Did not understand the seriousness of rabies 6. Lack of means for transportation |
| 3.28 If PEP was not taken, why was it not taken? | 1. Not aware of the need to get PEP 2. PEP not given/advised by the hospital 3. ARV was not available at the hospital during the visit 4. Bitten by owned dog and the dog was vaccinated against rabies 5. Hospital far away and didn’t get time to visit the hospital 6. Sought local/religious treatment 7. Did not know biting animal would be rabid 8. Animal was normal 9. Waited for 10day observation 10. Considered exposure to be minor and no risk 11. No means for transportation 12. Do not like attitudes of hospital staffs 13. Fear of Injection 14. Clinician told not to come for the last dose |
| 3.29 Did you receive any injection at the site of bite wound? *(To understand whether RIG injection was given or not).*  *(Extract from ARV register)*  **NA for exposed case who did not seek PEP* | 1. Yes 2. No |

**Part IV: Cost associated in seeking PEP**

| 4.1 How far is/was the nearest PEP centre from your place? | 1. Less than 1 km 2. 2 to 5 km 3. 5 to 10 km 4. 10 to 15 km 5. More than 15km |
| --- | --- |
| 4.2 How did you travel to the hospital to get medical advice/PEP? | 1. Walk 2. Own car 3. Friends car 4. Taxi 5. Bus 6. Truck 7. Ambulance |
| 4.3 If you have travelled in Taxi/bus/truck to the hospital, how much was the approximate cost for each visit to get PEP *(including to and fro journey for each visit)?*  *During analysis, you have to account for the number of times visited—4 times* | 1. Less than Nu. 50 2. Nu. 50 to 100 3. Nu. 100 to 150 4. Nu. 150 to 200 5. Nu. 200 to 250 6. Nu.250 to 300 7. Nu. 300 to 500 8. Nu. 500 to 1000 9. More than 1000 |
| 4.4 How long did it take to visit the hospital, get PEP and then return back home for first time visit? | 1. Less than 1 hr 2. 1 to 3 hrs 3. 3 to 5 hrs 4. 5 to 8 hrs 5. 2 days |
| 4.5 How long did it take to visit the hospital, get PEP and then return back home during subsequent follow up visit? | 1. Less than 1 hr 2. 1 to 3 hrs 3. 3 to 5 hrs 4. 5 to 8 hrs 5. 2 days |
| 4.6 Did your family members, neighbour or friend accompany you during visit to the hospital? *(this is in case of an adult victim)* | 1. Yes 2. No |
| 4.7 If yes, did he/she travel all 4 times along with you to the hospital? | 1. 1 time 2. 2 times 3. 3 times 4. 4 times |
| 4.8 What is the occupation of the person who accompanied you to the hospital/BHU? | 1. ☐ Farmer 2. ☐ Student 3. ☐ House wife 4. ☐ Govt. employee 5. ☐ Businessman 6. ☐ Private/corporate sectors 7. ☐ Pre-school 8. ☐ Military 9. ☐ Monks/Nuns 10. ☐ Others |
| 4.9 Have you been admitted to the hospital due to bite? | 1. Yes 2. No |
| 4.10 If yes, how many days were you admitted? | 1. 1 day 2. 2 days 3. 3 days 4. 4 days 5. 5 days 6. More than 5 days |

**Part V: Patient outcome**

| 5.1 What is outcome of the animal exposed victims *(If the victim is dead, his/her family will be interviewed)* | 1. Alive and normal 2. ill with rabies suggestive symptoms (Hydrophobia, aerophobia, photophobia, paralysis or hyper excitability) 3. Dead |
| --- | --- |
| Have the victim received rabies PEP? | 1. ☐ Yes  2. ☐ No |
| 5.2 If dead or ill, what were the clinical sign and symptoms shown by the victim? | 1. Altered sensation at bite site 2. Fever 3. Nausea and vomiting 4. Hydrophobia 5. Aerophobia 6. Photophobia 7. Hyperexcitability 8. Excessive salivation 9. Confusion 10. Paralysis of lower limbs |
| 5.3 If dead or ill, how many days after animal exposure did victim show clinical sign and symptoms?  *(Correlate with date of exposure)* | Number of days: ……………………………………………………. |
| 5.4 Where did the victim die? | 1. Home 2. Hospital 3. Others |
| 5.5 If the death was at home, have the death been notified to the health officials? | 1. Yes 2. No |
| 5.6 If the death was at home, what were the reasons for not taking the victim to the hospital? | 1. Consulted local healers or performed ritual 2. Local healers did not approve taking to hospital 3. Rabies was confirmed at the hospital and since   there was no cure, victim was discharged |
